# Diagnostic value of selected fetal echocardiographic parameters in the prenatally suspected bicuspid aortic valve

**DOI:** 10.1186/s44156-024-00065-w

**Published:** 2025-01-06

**Authors:** Min Zheng, Yanping Ruan, Lin Sun, Xiaowei Liu, Jiancheng Han, Yihua He

**Affiliations:** 1 Echocardiography Medical Center, Beijing Anzhen Hospital, Capital Medical University, 2 Anzhen Road, Chaoyang District, Beijing, 100029 China; 2 Beijing Key Laboratory of Maternal-Fetal Medicine and Fetal Heart Disease & Echocardiography Department, Beijing Anzhen Hospital, Capital Medical University, Beijing, China

**Keywords:** Congenital heart disease, Bicuspid aortic valve, Fetus, Echocardiography, Follow up

## Abstract

**Objective:**

To explore the diagnostic value of crucial parameters of echocardiography for fetal bicuspid aortic valve (BAV) and improve diagnostic accuracy.

**Methods:**

Fetuses with a prenatal suspected diagnosis of BAV were followed, and confirmed and misdiagnosed cases were obtained. Prenatal echocardiography was reviewed and analyzed. ROC curves were plotted to evaluate the diagnostic capabilities of different echo signs.

**Results:**

14 cases were confirmed, and 7 patients were misdiagnosed. Some abnormal ultrasound signs were observed in both groups, including direct ultrasound signs of the aortic valve: Two commissures and a “fish-mouth” opening; Thickening, hyperechogenicity, or the presence of a raphe; Restricted motion or opening; Eccentric or a-linear valve leaflet closure line and indirect ultrasound signs: Increased supra-aortic valve velocity; Post-stenotic widening of the ascending aorta. The combination of “Increased supra-aortic valve velocity” and “Two commissures and a ‘fish-mouth’ opening” had the highest AUC (AUC: 0.893, 95%CI: 0.752-1.000, Sensitivity: 0.786, Specificity: 1.000).

**Conclusions:**

We first found that the combination of “Increased supra-aortic valve velocity” and “Two commissures and a ‘fish-mouth’ opening” had the best diagnostic capability and could reduce the rate of misdiagnosis. Fetuses with BAV should be followed up prenatally for the aortic valve and ascending aorta as they progressively deteriorate with gestational age.

## Introduction

Bicuspid aortic valve (BAV) is one of the most common congenital heart abnormalities, with an incidence of about 0.5-2%, and is more common in men [1]. BAV can occur as an isolated disease or in combination with other heart defects and coarctation of the aorta, particularly [2, 3]. It is relatively simple to display the aortic valves in children and adults by echocardiography. Many other imaging modalities are available, so BAV has been extensively and deeply studied in the pediatric and adult fields. The morphology, classifications, Genesis, and complications of BAV were also thoroughly researched [4–6].

The diagnosis of fetal heart disease mainly relies on fetal echocardiography. Owing to the size of the heart and the decreased conditions for examination inside the mother’s uterus, it is difficult to correctly diagnose the BAV prenatally. There are few related studies [3, 7]. D. PALADINI et al. demonstrated that aortic valve anatomy could be satisfactorily assessed in fetuses. They believed that a detailed scan for a BAV should be attempted in all patients with a positive family history of congenital heart disease, particularly of left ventricular outflow tract obstruction or bicuspid aortic valve [3]. Nevertheless, there are no criteria for diagnosing fetal BAV, and sonographers sometimes suspect BAV when abnormal aortic valve function is observed, leading to a high rate of misdiagnosis. Parents may choose to induce labor as a result.

In this study, we followed up fetuses with a prenatal suspected diagnosis of BAV in our center, obtained groups of confirmed cases and misdiagnosed cases after birth, and analyzed the prenatal echocardiographic characteristics of both groups, aiming to find critical parameters that can help diagnose fetal BAV and to improve the diagnostic accuracy.

## Materials and methods

### Population

This study was conducted in Beijing Anzhen Hospital, and patients with a prenatal suspected diagnosis of BAV were followed up between January 2017 and April 2022. All patients had no combined congenital heart disease other than aortic disease. A group of confirmed cases and a group of misdiagnosed cases were obtained. Baseline demographics and prenatal echocardiography were reviewed and analyzed for both groups of cases. All the patients signed the consent forms, and the institutional ethics committee of Beijing Anzhen Hospital approved this study.

### Examination

Echocardiography is an effective screening method for the fetus. All ultrasound examinations were performed by The General Electric Voluson E8 or E10 ultrasound system with transabdominal 2- to 4-MHz curvilinear transducers (GE Healthcare Ultrasound, Milwaukee, WI, USA). Based on the guidelines of the American Society of Echocardiography [8], for each gravida referred to this center, an exhaustive fetal echocardiographic examination was made, including two-dimensional (2D), M-mode, color, and pulse Doppler. In addition, some extracardiac echo data were also obtained to confirm if the fetus had a standard growth curve or abnormalities.

In the left ventricular outflow tract view, if suspicious ultrasound signs of the aortic valves were observed, such as thickening, hyperechogenicity, restricted motion or opening, eccentric closure line, increased supra-aortic valve velocity, or the dilated ascending aorta, the short axis of the right ventricle should be observed to determine the number of the commissures, raphe, a linear or Y shape leaflet closure line and arrangement of the aortic valve leaflets. The images could be magnified to visualize the valve. The width of the ascending aorta can be obtained from the left ventricular outflow tract view. The echo data were re-evaluated by two experienced fetal cardiologists who were blinded.

### Follow up

Clinical follow-ups were implemented via a standardized telephone call by a trained nurse or having patients reviewed on an outpatient basis, which focused on pregnancy outcomes (intrauterine death, labor induction, autopsy, birth), postnatal findings (echocardiography, magnetic resonance imaging, cardiac catheterization, etc.), child growth and development. The aortic valve leaflets and aorta conditions were carefully inquired, such as the degree of stenosis or regurgitation, with or without other malformations or gene abnormality.

### Statistical analysis

Continuous variables are reported as the mean, median, and interquartile range (IQR). Categorical variables are reported as proportions. This work plotted receiver operating characteristic (ROC) curves to evaluate different echo signs’ diagnostic capabilities. IBM SPSS Statistics (Version 21.0) was used to analyze the data. A value of *P* < 0.05 was considered statistically significant.

## Results

After a follow-up visit, it was found that 14 cases were confirmed with BAV, and 7 were misdiagnosed (6 cases showed normal tricuspid aortic valve, and 1 case had aortic valve calcification and stenosis). The clinical information and prenatal ultrasound findings were summarized in Table 1, including 1 case of twins and 20 cases of a singleton. Among the 14 confirmed cases, 10 are males, which showed a male predominance. The mean maternal age at diagnosis was 30.5 ± 4.7 years old. None of their parents had a history of congenital heart disease. One of them had a risk factor of getting a cold during pregnancy. They only underwent non-invasive genetic testing, and no abnormalities were found.

**Table 1:** Clinical information, fetal echocardiographic characteristics, and outcome of 14 confirmed cases and 7 misdiagnosed cases

Case	Sex	Gestational week	Direct echo signs				Indirect echo signs			Postnatal outcome
			Two commissures and a “fish-mouth” opening	Thickening, hyperechogenicity, or the presence of a raphe	Restricted motion or opening	Eccentric or a-linear closure line	Supra-aortic valve velocity (cm/s)	Ascending aorta width (mm)	Z score of the ascending aorta	
1	F	27 + 2	-	+	+	+	143	4.2	-1.8	BAV, 177 cm/s
2	M	23 + 0	-	-	+	-	280	5.5	2.5	BAV
3	M	28 + 0	-	-	-	-	125	7.7	3	BAV, 192 cm/s, no raphe
4	M	27 + 6	+	-	-	-	128	6.7	2.2	BAV, 265 cm/s, one raphe
5	F	22 + 3	+	-	+	+	119	5	2	BAV, 334 cm/s, no raphe
6	M	27 + 1	+	-	-	+	103	5.7	1.2	BAV
7	M	29 + 4	+	+	-	-	200	4.7	1.1	BAV, 284 cm/s, one raphe
8	F	27 + 3	+	+	-	-	139	5	-1.1	BAV, 225 cm/s, one raphe
9	M	24 + 1	+	+	-	-	114	5.9	2.4	BAV
10	M	27 + 2	+	+	-	-	102	8.1	3.9	BAV, 158 cm/s, no raphe
11	M	23 + 1	+	+	+	-	136	6	3	BAV, 259 cm/s, one raphe
12	F	27 + 3	+	+	+	-	266	7.5	3.2	BAV, aortic valve intervention
13-first	M	25 + 4	+	+	+	-	110	6	2.4	BAV
13-second		30 + 4					148	7.4	2.4	
13-third		34 + 2					158	8.4	1.8	
14-first	M	21 + 3	+	+	+	-	140	4.5	1.1	BAV
14-second		25 + 5					182	7	3.3	
14-third		27 + 6					221	7.7	2.8	
15	F	25 + 0	-	-	-	-	105	5	0.6	TAV
16	M	29 + 4	-	-	-	-	121	7.5	2.5	TAV
17	M	26 + 6	+	-	-	-	76	6.2	2.1	TAV
18	M	25 + 6	+	-	-	-	75	4.5	1	TAV
19	F	25 + 1	-	-	-	-	117	4.6	0	TAV
20	F	29 + 6	+	+	+	-	93	5	-0.68	TAV
21	F	24 + 1	-	+	+	-	239	6	2.7	TAV, calcification

BAV, bicuspid aortic valve (Case1 to 14); TAV, tricuspid aortic valve (Case15 to 21); F, female; M, male

**Fig. 1:**
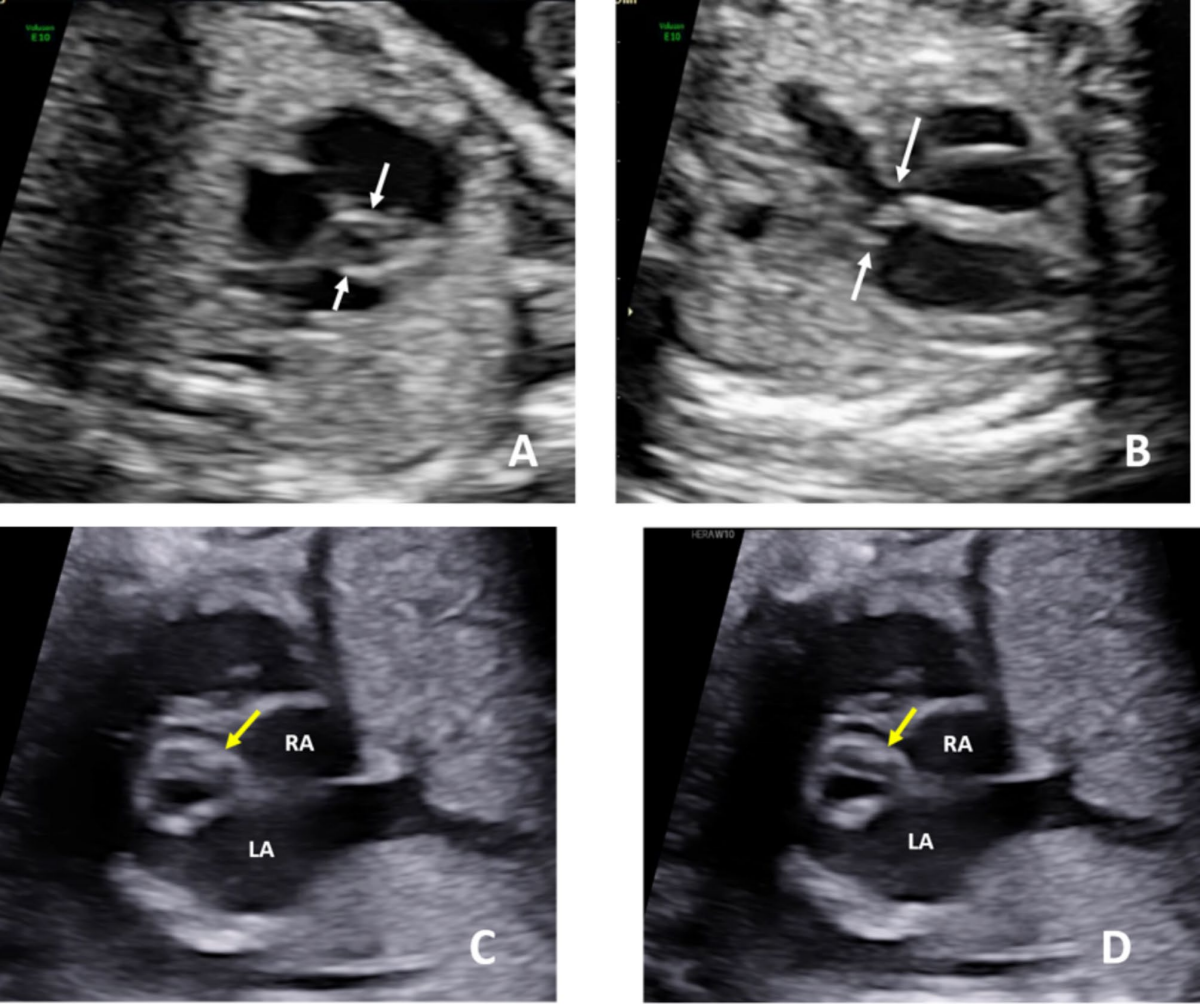
Bicuspid aortic valve in fetal 2-dimensional echocardiogram: The short axis view of the right ventricle shows two commissures and a “fish-mouth” opening (**A**). The left ventricular outflow tract view shows thickening and hyperechogenicity (**B**). The short axis view of the right ventricle shows a raphe (**C** and **D**)

**Fig. 2:**
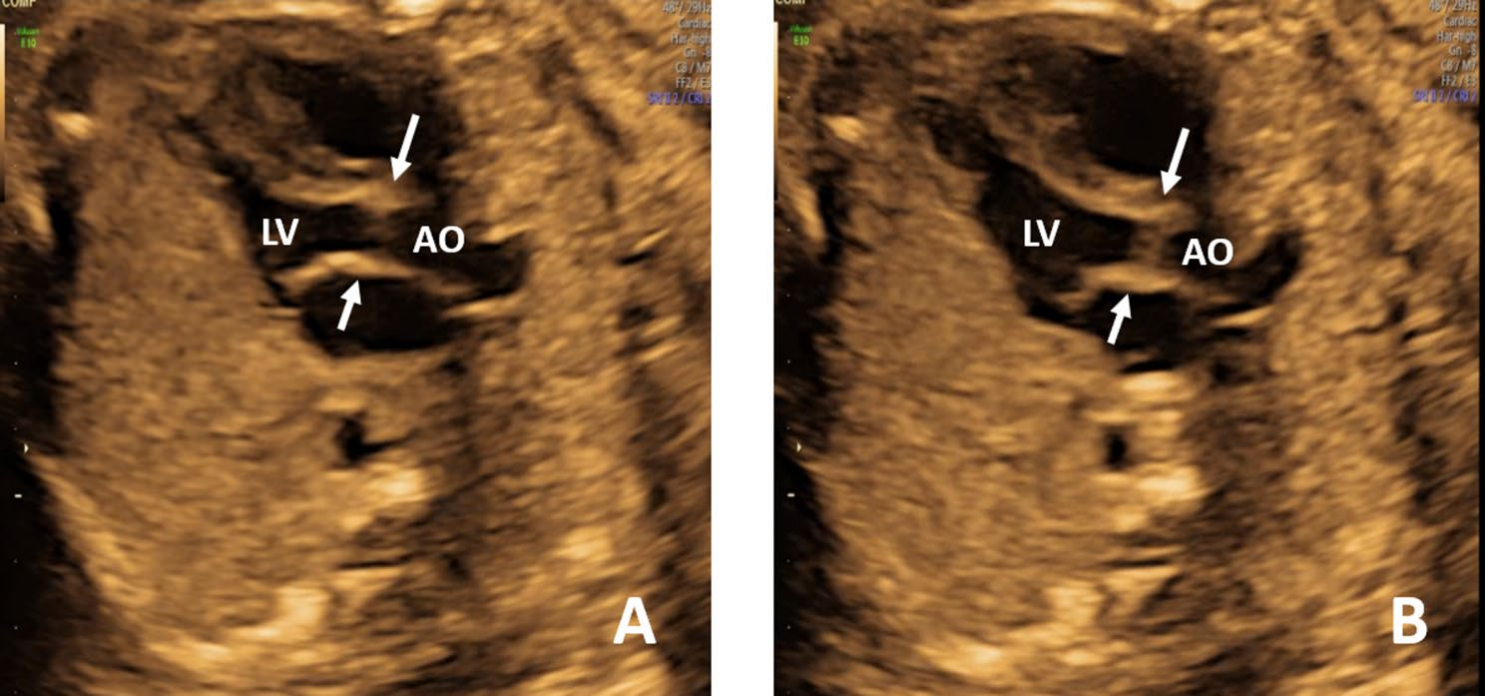
The left ventricular outflow tract view. The restricted activity of the bicuspid aortic valve both in systole (**A**) and diastole (**B**). LV, left ventricle; AO, ascending aorta

**Fig. 3:**
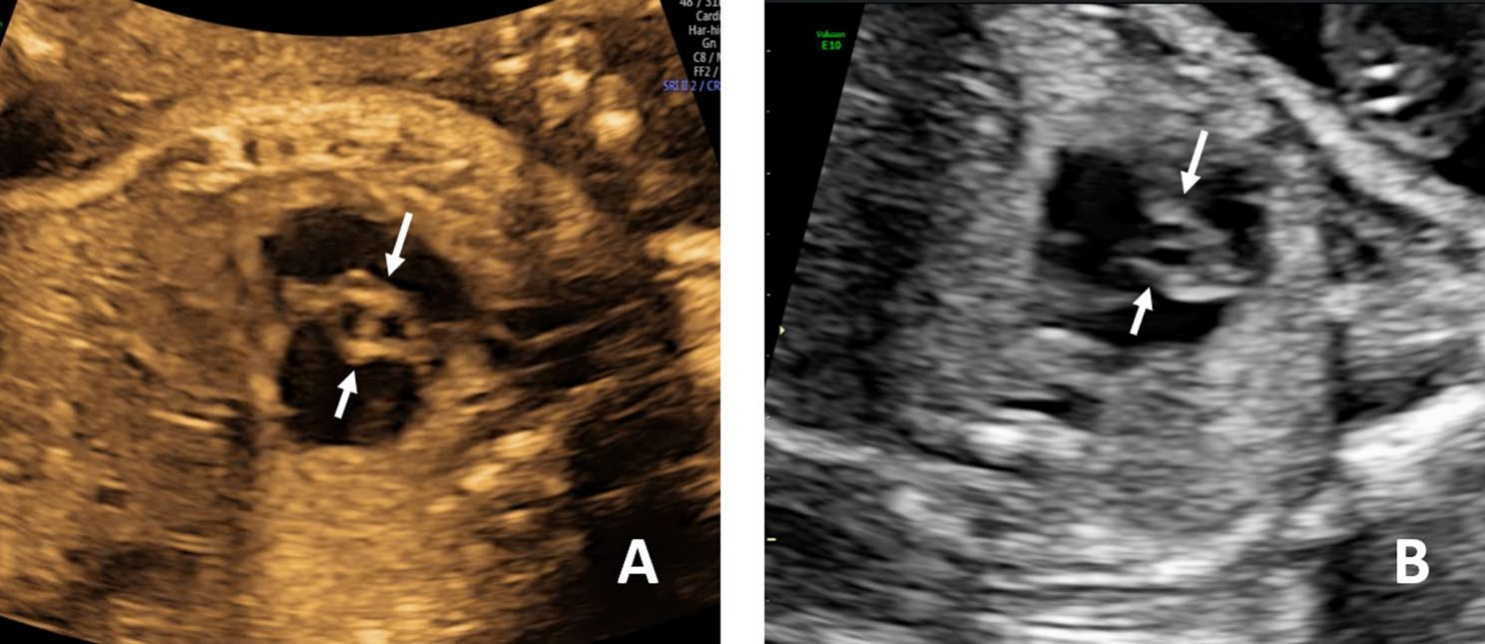
The short axis view of the right ventricle showing one-line valve leaflet closure line (**A** and **B**)

**Fig. 4:**
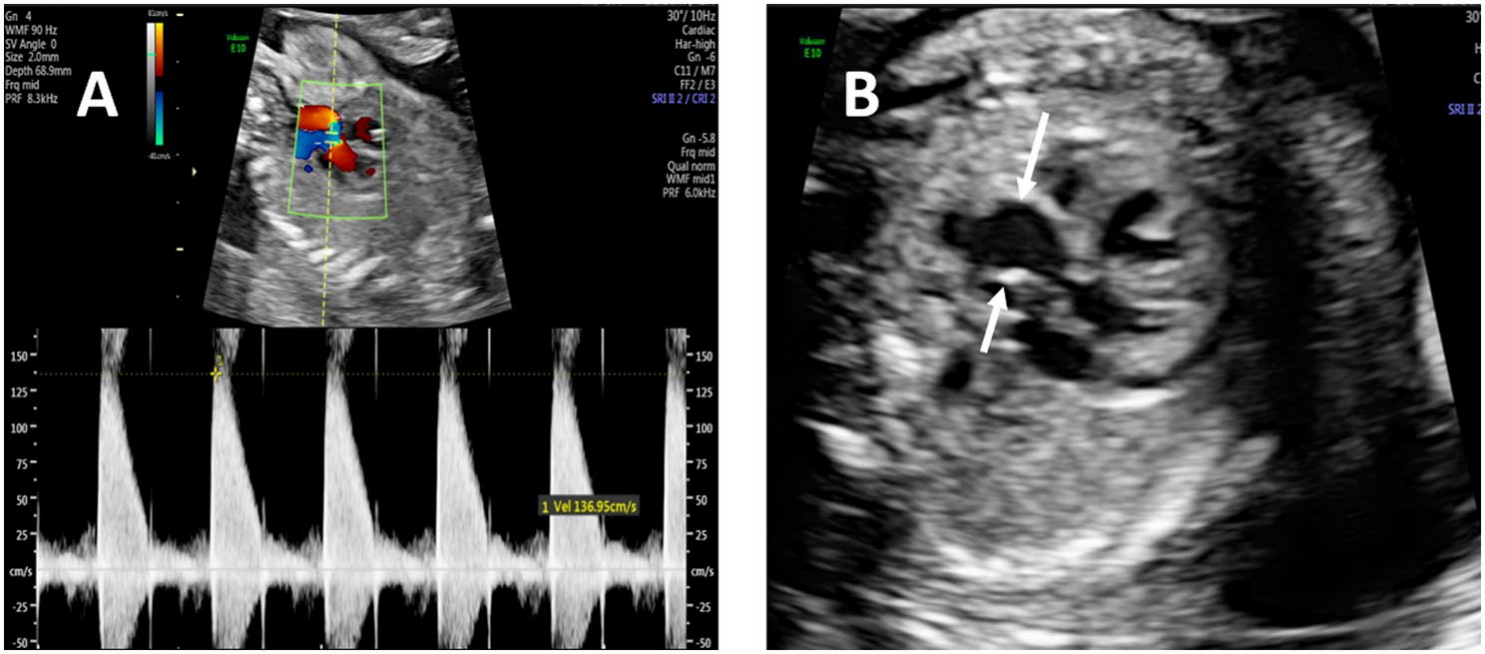
Indirect ultrasound signs of the fetal bicuspid aortic valve. Increased supra-aortic valve velocity (**A**). Post-stenotic widening of the ascending aorta (**B**)

When we reviewed the fetal echocardiograms, we found that some abnormal ultrasound signs could be observed in confirmed and misdiagnosed cases, indicating possible aortic valve abnormalities. And we divided these signs into direct and indirect echocardiographic signs, as shown in Table 1. Direct ultrasound signs of the aortic valve included two commissures and a “fish-mouth” opening (Fig. 1a); thickening, hyperechogenicity, or the presence of a raphe (Fig. 1b, c and d); restricted motion or opening (Fig. 2); eccentric or a-linear valve leaflet closure line (Fig. 3). Indirect ultrasound signs included increased supra-aortic valve velocity (above 100 cm/s [9], Fig. 4a); post-stenotic widening of the ascending aorta (Z score greater than 2 points, Fig. 4b). The percentage of direct and indirect ultrasound signs in confirmed and misdiagnosed cases was summarized in Table 2. Increased supra-aortic flow velocity was present in all confirmed cases (14 cases, 100%), suggesting fetal aortic stenosis or potential stenosis in this group of cases. Aortic regurgitation was not observed prenatally in any case, and only Case 3 had been observed with mild regurgitation from 20 months after birth.

**Table 2:** Percentage of direct and indirect ultrasound signs in confirmed and misdiagnosed cases

	Confirmed 14 cases	Misdiagnosed 7 cases
**Direct ultrasound signs**		
Two commissures and a “fish-mouth” opening	11 cases, 78.6%	3 cases, 42.9%
Thickening, hyperechogenicity, or the presence of a raphe	8 cases, 57.1%	2 cases, 28.6%
Restricted motion or opening	6 cases, 42.9%	2 cases, 28.6%
Eccentric or a-linear closure line	2 cases, 14.3%	0 cases, 0%
**Indirect ultrasound signs**		
Increased supra-aortic valve velocity	14 cases, 100%	4 cases, 57.1%
Post-stenotic widening of the ascending aorta	10 cases, 71.4%	3 cases, 42.9%

**Fig. 5:**
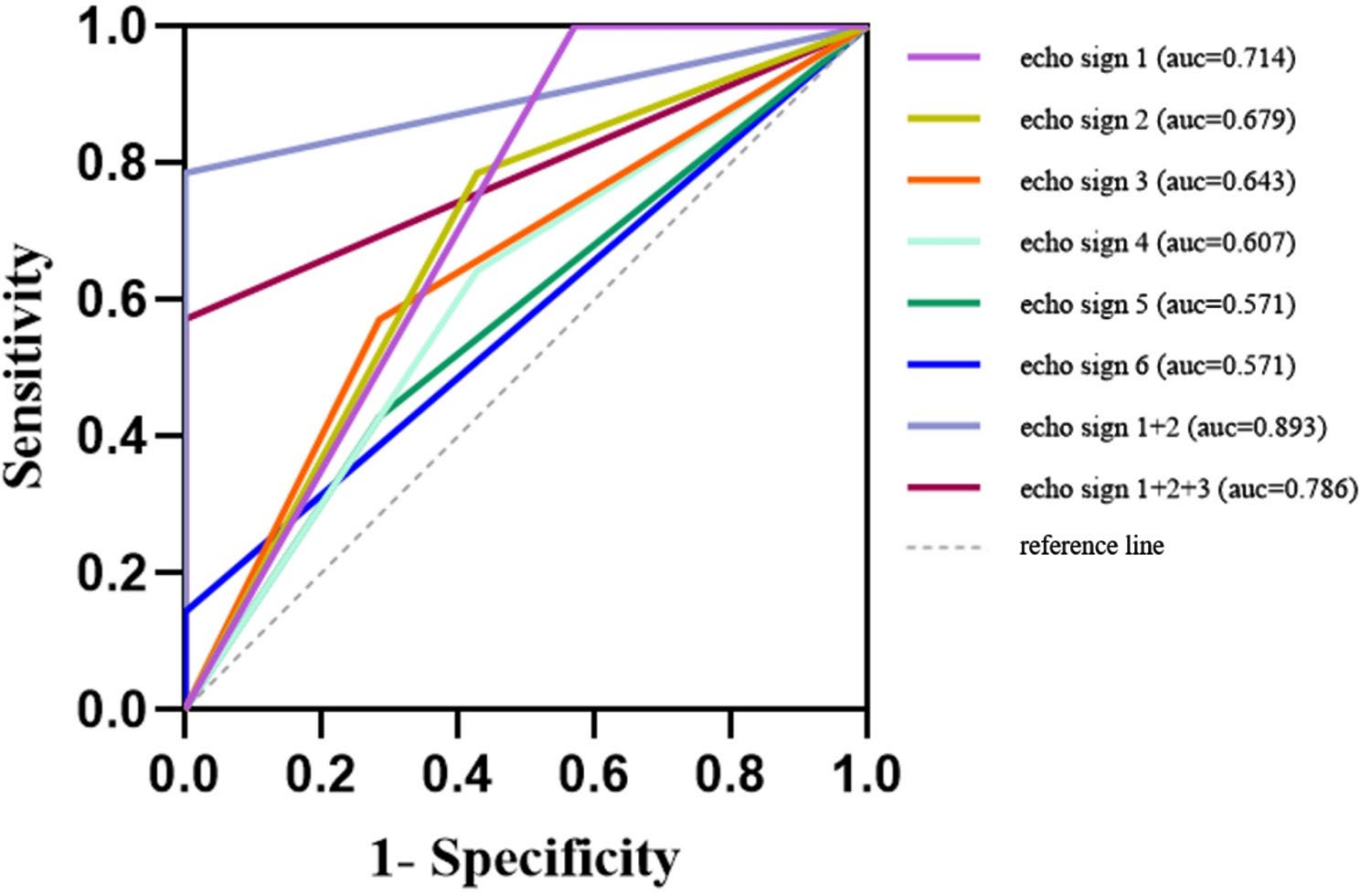
ROC curves of different echo signs. The AUC of echo sign 1 was the highest, followed by echo signs 2 and 3. The AUC was significantly increased for the combination of echo signs 1 and 2. When echo signs 1, 2, and 3 were combined, the AUC decreased but was higher than echo sign 1. Echo sign 1, Increased supra-aortic valve velocity; echo sign 2, Two commissures and a “fish-mouth” opening; echo sign 3, Thickening, hyperechogenicity, or the presence of a raphe; echo sign 4, Post-stenotic widening of the ascending aorta; echo sign 5, Restricted motion or opening; echo sign 6, Eccentric or a-linear closure line

The diagnostic value of all echogenic signs for fetal BAV was analyzed by ROC. The area under the curve (AUC), sensitivity, and specificity were presented in Table 3. As shown in Fig. 5, the AUC of “Increased supra-aortic valve velocity” (echo sign 1) was the highest (AUC:0.714, 95%CI:0.450–0.979, Sensitivity:1.000, Specificity:0.429), followed by “Two commissures and a ‘fish-mouth’ opening” (echo sign 2, AUC: 0.679, 95%CI:0.421–0.936, Sensitivity: 0.786, Specificity: 0.571) and “Thickening, hyperechogenicity, or the presence of a raphe” (echo sign 3, AUC:0.643, 95%CI:0.390–0.896, Sensitivity:0.571, Specificity:0.714). The AUC was significantly increased for the combination of echo signs 1 and 2 (AUC: 0.893, 95%CI: 0.752-1.000, Sensitivity: 0.786, Specificity: 1.000). When we combined echo signs 1, 2, and 3, the AUC decreased (AUC: 0.786, 95%CI: 0.593–0.979, Sensitivity: 0.571, Specificity: 1.000), but also higher than echo sign 1.

**Table 3:** Diagnostic capability of different echo signs

Number	Echo signs	AUC (95% confidence interval)	Sensitivity	Specificity	*p*
1	Increased supra-aortic valve velocity	0.714(0.450–0.979)	1.000	0.429	0.117
2	Two commissures and a “fish-mouth” opening	0.679(0.421–0.936)	0.786	0.571	0.192
3	Thickening, hyperechogenicity, or the presence of a raphe	0,643(0.390–0.896)	0.571	0.714	0.296
4	Post-stenotic widening of the ascending aorta	0.607(0.344–0.870)	0.643	0.571	0.433
5	Restricted motion or opening	0.571(0.310–0.833)	0.429	0.714	0.602
6	Eccentric or a-linear closure line	0.571(0.317–0.826)	0.143	1.000	0.602
1 + 2		0.893(0.752-1.000)	0.786	1.000	0.004
1 + 2 + 3		0.786(0.593–0.979)	0.571	1.000	0.037

AUC = area under the receiver operating characteristic curve

Cases 13 and 14 both underwent three times of fetal echocardiograms at our center, with an interval of 2–4 weeks. It was found that with increasing gestational age, the supra-aortic valve velocity gradually increased, and the ascending aorta gradually dilated.

Two confirmed cases (14.3%, case 5 and case 12) had growth restrictions and feeding difficulties after birth. Case 5 was a dizygotic twin pregnancy. One of the fetuses had BAV, while her twin sister had a tricuspid aortic valve. After birth, she had a repeat echocardiogram at two months, three months, and two years of age. Her supra-aortic valve velocity was all greater than 330 cm/s, representing a moderate degree of stenosis. When they came for review at age three, the child with BAV was significantly behind in growth and development. Due to symptoms and moderate aortic valve stenosis, the pediatric surgeon recommended an aortic valve intervention. Case 12 received aortic valve balloon valvuloplasty at two months due to severe aortic valve stenosis. The rest of the patients grew and developed well.

## Discussion

BAV is a relatively common cardiac malformation, which accounts for 1.96% of the population of fetuses with congenital heart disease diagnosed in our center. Many patients present with infective endocarditis or aortic dissection at the initial diagnosis, and it may even be life-threatening [10]. Therefore, early diagnosis is extremely important, which can prompt patients to adopt a reasonable lifestyle and regularly follow up on the aortic valve to avoid delayed treatment.

To our knowledge, prenatal screening for BAV is not widely available in any country, and screening for BAV has been suggested for high-risk patients with a family history of BAV [11]. There is no evidence in the literature on how to accurately screen fetuses with BAV and abnormal aortic valves. This study provides some clues for accurately screening such patients, indicating that fetuses with BAV and aortic stenosis have some direct and indirect echocardiographic signs prenatally. They can appear alone or in combination of multiple signs.

After using ROC analysis of each ultrasound sign, we found that the diagnostic capability of a single parameter was low. The highest AUC was found for “Increased supra-aortic valve velocity,” but the diagnostic specificity was only 0.429. The combination of “Increased supra-aortic valve velocity” and “Two commissures and a ‘fish-mouth’ opening” had the best diagnostic capacity, superior to the three parameters combined. Therefore, when supra-aortic valve velocity above 100 cm/s is prenatally observed in the left ventricular outflow tract view, the short-axis view of the aortic valve should be scanned to determine if the aortic valve has two commissures and a “fish-mouth” opening. When both ultrasound signs are present, fetal BAV can be diagnosed. The sensitivity is 0.786 and the specificity is as high as 1. This finding can guide ultrasound doctors to accurately diagnose BAV prenatally and reduce the misdiagnosis rate.

Conventional fetal echocardiography may miss fetuses with BAV. Because in the latest 2023 ISUOG fetal echocardiography guidelines [12], the short-axis view of the aortic valve is not a routine scanning view. If there is no obvious abnormality in the aortic valve function of the patient, it may be missed. Secondly, fetal echocardiography is generally scanned in the second trimester. And we observe that the valve function of BAV may gradually deteriorate as the gestational age increases. So some patients with problems only emerging in the third trimester may also be missed. Furthermore, for some patients with poor acoustic windows, accurately scanning the morphology of the fetal aortic valve is challenging. Therefore, this article suggests that if some suspicious BAV ultrasound signs are found during the routine fetal echocardiography scan in the second trimester, pregnant women can be advised to recheck fetal echocardiography in the third trimester. This can help observe the degree of valve and aortic lesions and identify some patients who were missed in the second trimester.

BAV disease is typically associated with aortic valve stenosis, aortic valve regurgitation, and ascending aorta dilatation [7, 13]. A previous study found that 37% of fetuses with dilated ascending aorta were associated with BAV. In this study, aortic valve stenosis and dilatation of the ascending aorta were observed in most diagnosed cases. Only one patient developed aortic valve regurgitation during follow-up. The pathogenesis of dilated ascending aorta includes the “genetic theory” and the “hemodynamic theory”, which are not contradictory. The genetic theory posits that mutations in different genes in BAV patients simultaneously affect the aorta as the aortic valve and ascending aorta share the same embryological origin. Alejandro Junco-Vicente believed that several genetic mutations can only explain less than 5–10% of BAV cases [14, 15]. The hemodynamic theory suggests that the anomalous opening of BAV leads to an eccentric flow directed against the aortic wall. The constant tangential force generates the widening, known as “wall shear stress” (WSS) [16]. None of the patients in this study underwent genetic analysis. Two of the confirmed patients underwent three times of fetal echocardiographic examinations, showing that the supra-aortic valve velocity and the width of the ascending aorta increased with gestational age. In the early stage, the width of the ascending aorta could be within the normal range, so it is speculated that the widening of the ascending aorta may be related to the constant tangential force caused by BAV since its impact is time-dependent. These two cases also remind us that the degree of prenatal aortic valve stenosis is not static and will gradually increase with gestational age. The degree of stenosis after birth may be more consistent with that in late pregnancy.

Most patients with BAV have normal aortic valve function during the fetal period and have a good prognosis, so early screening is not required. However, some patients who have abnormal aortic valve function (mainly stenosis) during the fetal period need to be detected as early as possible. If the patient has severe aortic valve stenosis, when the patent ductus arteriosus (PDA) of some newborns closes, acute cardiogenic shock may occur, manifested as hypotension, poor peripheral perfusion and cyanosis, and urgent aortic valve surgery intervention is required. Aortic valve stenosis in infancy can be manifested as congestive heart failure, such as restricted growth and development, feeding difficulties and shortness of breath. Older children and adolescents rarely have symptoms and are usually diagnosed during physical examinations. Some children with severe aortic valve stenosis will show easy fatigue, shortness of breath, syncope, chest pain or angina when exercising. When children have related symptoms, a reexamination should be carried out immediately [6]. Therefore, it is recommended that all patients with suspected BAV prenatally undergo fetal echocardiography reexamination in the third trimester to follow up the degree of aortic valve lesions. If obvious stenosis or regurgitation occurs, it is recommended to recheck as soon as possible within three days after birth, and at most no more than one week. For other children, echocardiography can be rechecked within half a year after birth.

Previous studies have shown that about 12-15% of patients need intervention treatment before adulthood. More than half of BAV patients need aortic valve replacement during their lifetime, and more than 25% of patients need aortic replacement [6, 17–19]. In this study, a confirmed patient underwent intervention in the early stage after birth due to aortic valve stenosis. The flow velocity above the aortic valve was 266 centimeters/second at 27 weeks of gestation, indicating severe stenosis. Therefore, the discovery of high aortic valve flow velocity prenatally may have a suggestive effect on the appearance of symptoms in the early stage after birth. The discovery of BAV during the fetal period is helpful to guide patients to have regular follow-up after birth and avoid delayed treatment and growth restriction [3].

At present, some evidence shows that early drug treatment can significantly prolong the asymptomatic time of patients with BAV or postpone the need for surgical treatment. First, patients with bicuspid aortic valve and hypertension should be actively treated, because hypertension is a risk factor for aortic dilation. Secondly, the current guidelines recommend routine antibiotic prophylaxis before dental or invasive respiratory procedures for BAV patients who have a history of endocarditis attack or who have undergone aortic valve surgery and replacement [6]. Thirdly, if patients have relevant symptoms such as heart failure, they should also receive drug treatment as soon as possible. This can postpone the timing of surgical treatment to a certain extent. However, although drugs may help control symptoms and slow down the progression of the disease to a certain extent in some cases, they are unlikely to completely stop or reverse the underlying valve abnormalities.

In conclusion, we first explored the key ultrasound parameters for diagnosing fetal BAV. We found that the combination of “Increased supra-aortic valve velocity” and “Two commissures and a ‘fish-mouth’ opening” had the best diagnostic capability and could reduce the rate of misdiagnosis. Fetuses diagnosed with BAV should be followed up prenatally for the aortic valve and ascending aorta as they progressively deteriorate with gestational age.

## Limitations

Our study had several limitations: (1) The subjects enrolled in this study were fetuses with suspected BAV, and all had abnormal prenatal ultrasound; for BAV fetuses with normal prenatal ultrasound, they were not routinely scanned for short-axis views of the great arteries, so these fetuses were missed. And there was selection bias in the study subjects, and the clinical data were not representative of the entire BAV population. (2) The prenatal diagnosis of BAV is challenging. Although our center screens a large number of fetuses every year, the sample size of this study is still small. Logistic regression cannot be used to evaluate the probability of fetuses with suspected prenatal BAV being finally diagnosed with BAV. Therefore, we still need further large-scale prospective studies to verify the results of this study. (3) As the largest referral center for fetal heart disease in China, the accuracy of fetal echocardiography in diagnosing congenital heart disease is high, as confirmed by autopsy. However, there is still a high rate of misdiagnosis in fetuses with BAV, which proves the difficulty of prenatal diagnosis of this disease and requires more studies for exploration.

## Data Availability

No datasets were generated or analysed during the current study.
